# Plantar thermography predicts freedom from major amputation after endovascular therapy in critical limb ischemic patients

**DOI:** 10.1097/MD.0000000000022391

**Published:** 2020-11-13

**Authors:** Wei-Chun Chang, Chi-Yen Wang, Yutsung Cheng, Yu-Po Hung, Tzu-Hsiang Lin, Wei-Jhong Chen, Chieh-Shou Su, Chiann-yi Hsu, Tsun-Jui Liu, Wen-Lieng Lee

**Affiliations:** a Cardiovascular Center, Taichung Veterans General Hospital; b Department of Life Science, Tunghai University; c Biostatistics Task Force of Taichung Veterans General Hospital, Taichung; d Department of Medicine, National Yang-Ming University School of Medicine, Taipei, Taiwan.

**Keywords:** angiosome, multi-level intervention, infra-popliteal lesion, median runoff vessel numbers, plantar arch, thermal gradient

## Abstract

Although plantar thermography can evaluate the immediate perfusion result after an endovascular therapy (EVT) has been performed, a relevant wound outcome study is still lacking.

This study was to investigate whether angiosome-based plantar thermography could predict wound healing and freedom from major amputation after EVT in patients with critical limb ischemia (CLI)^[1]^.

All 124 patients with CLI (Rutherford category 5 and 6) who underwent EVT from January 2017 to February 2019 were prospectively enrolled. All patients received thermography both before and after EVT. Both wound healing and freedom from major amputation at the 6-month follow-up period were recorded. There were 61 patients in the healing group and 63 patients in the non-healing group, whereas the major amputation total was 14 patients. The mean pre- and post-EVT temperature of the foot was significantly higher in the healing group than in the non-healing group (30.78 °C vs 29.42 °C, *P* = .015; and 32.34 °C vs 30.96 °C, *P* = .004, respectively). DIFF2 was significantly lower in the non-healing group (-1.38 vs -0.90, *P* = .009). DIFF1 and DIFF2 were significantly lower in the amputation group (-1.85 °C vs -1.11 °C, *P* = .026; and -1.82 °C vs -1.08 °C, *P* = .004). Multivariate analysis showed that DIFF2 stood out as an independent predictor for freedom from major amputation (hazard ratio 0.51, *P* = .045). Receiver operating characteristic curve analysis showed a DIFF2 cut-off value of -1.30 °C, which best predicts freedom from major amputation.

Plantar thermography is associated with wound healing and helps predict freedom from major amputation in CLI patients undergoing EVT.

## 1 Introduction

Critical Limb Ischemia (CLI)^[1]^ represents the most advanced clinical form of peripheral artery disease (PAD). According to current guidelines, Endovascular Therapy (EVT) is considered an acceptable treatment for patients with CLI which is attributable to infra-popliteal lesions in certain patients.^[2,3]^ However, Iida et al reported 43.2% reintervention, 37% amputations, 43.2% wound recurrence and 37% all-cause mortality after EVT for infra-inguinal lesions in patients with CLI.^[4,5]^ Therefore, the ability to precisely predict outcomes for CLI patients after contemporary state-of-art EVT has been a long-awaited but unanswered issue in clinical practice.

Currently available tools for assessing EVT treatment outcomes, including angiography, the Ankle-Brachial Index (ABI), segmental limb pressure, toe pressure, Duplex ultrasound, skin perfusion pressure and Transcutaneous Pressure of Qxygen, have all shown significant limitations.^[6–9]^ On the contrary, limb temperature reflects blood flow secondary to vessel patency and may be a reasonable surrogate for EVT outcome. A decrease in temperature may indicate the presence of arterial occlusive disease and temperature changes in the lower limb can be indicative of diabetic complications such as ulceration.^[10,11]^ Thermography detects infra-red radiation emitting from the body region of interest, typically the skin, and presents the regional temperature as a heat zone image. Multiple studies have investigated the potential use of thermography in assessing peripheral perfusion and tissue viability.^[12–15]^ We have hypothesized that a change in foot temperature after EVT may be associated with wound healing and freedom from major amputation, and could also be used in predicting clinical outcomes even in ischemic limbs with concurrent infections. However, clinical trials for accessing the outcomes of EVT by infrared thermography,and using it for predicting wound healing and freedom from major amputation in CLI patients are still lacking.

The aim of this study was to explore the feasibility of using plantar thermography to assess reperfusion of the foot after EVT in CLI patients. Additionally, we sought to develop a possible thermographic parameter to predict short-term outcomes regarding healing and freedom from major amputations, in real-world practice.

## 2 Methods and materials

All consecutive patients diagnosed with CLI (Rutherford categories 5 and 6) who underwent EVT for multi-level lesions from January 2017 to February 2019 were prospectively enrolled. Patients with either acute occlusive or embolic limb ischemia, as well as unstable hemodynamic status were excluded. This study was approved by the ethics committee of Taichung Veterans General Hospital (Approval CE17317B), and followed the Declaration of Helsinki and the ethical standards of the responsible committee on human experimentation.

### 2.1 Endovascular intervention

All EVT planning and strategy were laid out based upon angiosome concept, and relevant lesions were set as treatment targets.^[16]^ The individual EVT procedures were left to the discretion of the treating interventionist and may target the common iliac, external iliac, superficial femoral, popliteal, tibial and/or peroneal arteries. After insertion of a guiding sheath, unfractionated heparin (100 U/kg) was administered. Adequate anticoagulation was achieved by assuring activated clotting time within 250 to 300 seconds. Dual antiplatelet therapy (aspirin at 100 mg/d and cilostazol at 200 mg/d, or clopidogrel at 75 mg/d) was initiated 1 week prior to EVT. Drug-eluting stents, drug-coating balloons and atherectomy devices could be used for femoropopliteal lesions, if necessary, available and affordable. The treatment result was assessed by an angiogram as well as the blood flow runoff grade both before and after intervention. Procedural success was achieved when the opening of at least 1 straight vessel down to the foot was obtained. After EVT, dual antiplatelet therapy was maintained for at least 3 months.

### 2.2 Infrared thermography

Infrared thermography was performed both before and after EVT. The subjects were allowed to rest in a room where the room temperature was controlled at 24 °C (to equilibrate body temperature at an ambient temperature). No body parts of the patient were close to or in contact with any hot or cold sources. The patients were also kept away from air convection sources. The local temperature of both the dorsal and plantar aspects of the foot was measured by a digital infrared thermal image system (Spectrum 9000-MB Series; United Integrated Service Co. Ltd, Taipei Hsien, Taiwan) with a temperature resolution of 0.05 K. An infrared thermal camera was positioned 1 meter away from the examination table, and a reference black plate that reflected the room temperature was placed beside the feet. The thermal images were recorded with the patient in a supine position, allowing for a 10-minute rest period after gauze was removed from the wound in the exam room. A high-resolution color image, which could be viewed on a miniature screen, was provided in real time. All images were standardized to the temperature range of 17 °C to 34 °C and converted to the rainbow color palette by the software. The surface temperature profiles were acquired and stored for subsequent analysis. The images were both obtained before EVT and on the day following EVT.

**Figure 1:**
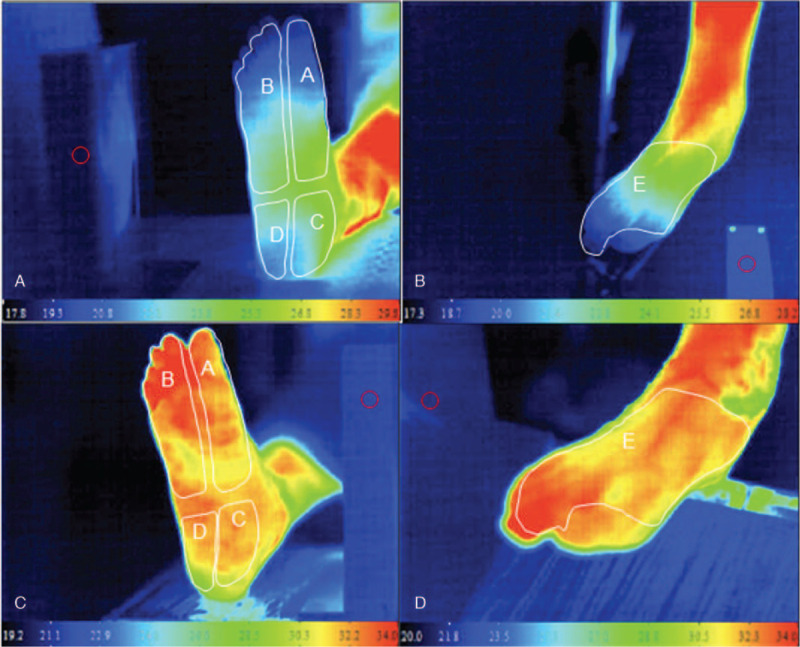
Typical thermal images of a patient's lower limb before and after successful EVT. Panel A: plantar aspect of right foot before EVT. The letters A-D depict angiosome-based thermographic zones used in this study. All zones, particularly in the digits, were hypothermic as compared with the normothermic zone in the lower leg. Panel B: Doral aspect of right foot. Letter E depicts zone of measurement in this study. It was also hypothermic, particularly in the digits. Panels C and D: Plantar and dorsal aspects of thermal images one day after successful EVT. All zones were normothermic.

In order to measure and analyze the regional temperature changes in the foot, the angiosome-based thermographic approach was adopted.^[17]^ As the open wound may be located at different aspects of the foot, thermal images of each foot were divided into 5 zones; one on the dorsal and 4 on the plantar aspects, corresponding to the angiosomes in the foot (Fig. 1). Mean pre-EVT temperature was defined as the mean of the temperatures in the 5 zones within 24 hours before EVT. Mean post-EVT temperature was defined as the mean of the 5 zone temperatures obtained within 24 hours after EVT. In order to develop a surrogate temperature for a better prediction on wound healing and freedom from major amputation, Difference Temperature 1 (DIFF1) and DIFF2 were calculated. DIFF1 was defined as the lowest temperature in any zone minus the mean pre-EVT temperature, whereas DIFF2 was defined as the lowest temperature minus the mean post-EVT temperature of the 5 zones.

### 2.3 Wound care and evaluation

Ischemic wounds were graded by following the University of Texas classification system, and were regularly managed by the PAD team and the plastic surgeons.^[18]^ Wounds were photographed and evaluated independently until they became completely healed, after which they were followed-up and assessed according to the standard protocol. Wound healing, defined as the complete epithelialization of the reference wound and possibly facilitated by surgical management (skin graft, flap, and suture closure) or secondary intervention, was recorded at 6 months (short-term result), and also regularly for up to a total follow-up period of 18 months. Major amputation was defined as above-ankle amputation and was recorded at 6 months (short-term result), and then regularly for up to a total follow-up period of 18 months.

### 2.4 Clinical follow-up

Clinical follow-ups were made at 1, 3, 6, 12, and 18 months, when each patient was assessed for ischemic presentations and thermography. Reintervention was initiated for the recurrence of rest pain, wounds or delayed wound healing. Patient survival, major amputation, re-intervention, and ulcer recurrence were confirmed by a telephone call if patients were unable to re-visit the hospital clinic.

### 2.5 Statistical analysis

Data analysis was performed by IBM SPSS version 22.0 (International Business Machines Corp, New York). Categorical data were expressed as frequencies or proportions and compared using the chi-square test or Fisher exact test, as appropriate. The normality of data distribution was checked using the Kolmogorov-Smirnov test. Because most values were not normally distributed, continuous variables were expressed as median values (first quartile, third quartile) and were compared by the Mann-Whitney *U* test. Receiver operating characteristic (ROC) curves were used to determine test sensitivity and specificity. The ultimate threshold value was determined by the maximum sum of sensitivity and specificity. Univariate and multivariate Cox regression analyses were performed to identify independent predictors for wound healing and freedom from major amputation. All statistical analyses were performed at a significance level of 2-sided *P* < .05.

## 3 Results

A total of 124 patients, 75 males and 49 females, with a median age of 73 (63-80) years old were recruited into this study. The patients’ baseline characteristics, angiographic findings and clinical outcome are presented in Tables 1 and 2. Ninety-five (95) (76.6%) of the patients had hypertension, 87 (70.2%) diabetes mellitus, 69 (55.6%) chronic kidney disease, and 43 (34.7%) were on regular hemodialysis. Most of the patients had infra-popliteal lesions (aortoiliac 9 (7.3%), femoropopliteal 76 (61.3%) and infra-popliteal 120 (96.8%)), and multi-level interventions were frequently performed. Amongst the infra-popliteal lesions, Chronic Total Occlusions were frequently present (101/124, 81.5%).

**Table 1:** Baseline demographic data.

	All patients N = 124	Healing in 6-M Group N = 61	Non-Healing in 6-M Group N = 63	*P* value
Age (yr)	73.0 (63–80)	71.0 (58–84)	73.0 (67–78)	.540
Gender-Male, N (%)	75 (60.5%)	42 (68.9%)	33 (52.4%)	.091
Body mass index, kg/m^2^	22.9 (19.8–26.4)	24.4 (20.1–26.9)	21.9 (19.4–26.1)	.180
Current smoker, N (%)	9 (7.3%)	6 (9.8%)	3 (4.8%)	.493
Hypertension, N (%)	95 (76.6%)	45 (73.8%)	50 (79.4%)	.601
Diabetes mellitus, N (%)	87 (70.2%)	40 (65.6%)	47 (74.6%)	.367
Hyperlipidemia, N (%)	32 (25.8%)	11 (18%)	21 (33.3%)	.082
Chronic lung disease, N (%)	13 (10.5%)	9 (14.8%)	4 (6.3%)	.217
Congestive heart failure, N (%)	44 (35.5%)	20 (32.8%)	24 (38.1%)	.667
Coronary artery disease, N (%)	44 (35.5%)	19 (31.1%)	25 (39.7%)	.421
Old myocardial infarction, N (%)	1 (0.8%)	0 (0.0%)	1 (1.6%)	1.000
History of CABG, N (%)	6 (4.8%)	2 (3.3%)	4 (6.3%)	.680
Previous PCI, N (%)	31 (25%)	14 (23%)	17 (27.0%)	.756
History of peripheral artery disease, N (%)	40 (32.3%)	16 (26.2%)	24 (38.1%)	.222
Previous lower limb bypass, N (%)	5 (4.0%)	2 (3.3%)	3 (4.8%)	1.000
Prior stroke or TIA, N (%)	17 (13.7%)	6 (9.9%)	11 (17.5%)	.331
Current dialysis, N (%)	43 (34.7%)	17 (27.9%)	26 (41.3%)	.168
Chronic kidney disease^∗^, N (%)	69 (55.6%)	28 (45.9%)	41 (65.1%)	.049
Atrial fibrillation, N (%)	18 (14.5%)	6 (9.8%)	12 (19.0%)	.230
Collagen vascular disease, N (%)	10 (8.1%)	4 (6.6%)	6 (9.5%)	.744
Creatinine (mg/dL)	1.4 (0.9–5)	1.2 (0.8–4.5)	1.8 (1–5.3)	.229
Estimated GFR (ml/min)	44.0 (10.4–84)	63.1 (10.2–88.9)	35.6 (10.5–74.9)	.185
HbA1C (mg/dL)	6.9 (6.1–8.7)	7.0 (6.2–9.2)	6.9 (6–8.4)	.333
LDL cholesterol, mg/dL	75.5 (56–101.8)	77.0 (59–105)	74.0 (54–101)	.612
Hemoglobin, g/dL	10.1 (8.6–11.7)	10.8 (8.9–12.9)	9.7 (8.5–10.7)	.004
ABI	0.8 (0.6–1)	0.8 (0.6–1)	0.8 (0.6–1)	.664
Rutherford classification, N (%)				<.001
Category 5	54 (43.5%)	37 (60.7%)	17 (27%)	
Category 6	70 (56.5%)	24 (39.3%)	46 (73.0%)	
University of Texas classification, N (%)				.057
Grade 0	11 (8.9%)	8 (13.1%)	3 (4.8%)	
Grade 1	43 (34.7%)	25 (41%)	18 (28.6%)	
Grade 2	29 (23.4%)	14 (23%)	15 (23.8%)	
Grade 3	41 (33.1%)	14 (23%)	27 (42.9%)	
Infection, N (%)	66 (53.2%)	30 (49.2%)	36 (57.1%)	.479
Medications,N (%)				
Aspirin	102 (82.3%)	52 (85.2%)	50 (79.4%)	.534
Clopidogrel	110 (88.7%)	52 (85.2%)	58 (92.1%)	.360
Cilostazol	80 (64.5%)	43 (70.5%)	37 (58.7%)	.238
Pentoxifylline	7 (5.6%)	4 (6.6%)	3 (4.8%)	.715
Warfarin or NOAC	11 (8.9%)	5 (8.2%)	6 (9.5%)	1.000
ACEI/ARB	54 (43.5%)	24 (39.3%)	30 (47.6%)	.454
Statin	74 (59.7%)	36 (59.0%)	38 (60.3%)	1.000
Beta blocker	38 (30.6%)	14 (23%)	24 (38.1%)	.102
Calcium channel blocker	61 (49.2%)	32 (52.5%)	29 (46.0%)	.592
Insulin	32 (25.8%)	16 (26.2%)	16 (25.4%)	1.000
Prostaglandin E1	35 (28.2%)	19 (31.1%)	16 (25.4%)	.609

**Table 2:** Lesion characteristics and clinical outcomes.

	All Patients N = 124	Healing in 6-M Group N = 61	Non-Healing in 6-M Group N = 63	*P* value
Aortoiliac lesion, N (%)	9 (7.3%)	1 (1.6%)	8 (12.7%)	.033
TASC II classification				1.000
TASC C	2 (22.2%)	0 (0%)	2 (25%)	
TASC D	7 (77.8%)	1 (100%)	6 (75%)	
CTO	4 (3.2%)	1 (1.6%)	3 (4.8%)	.619
Vessel calcification^∗^				.268
Grade 2	2 (22.2%)	0 (0%)	2 (25%)	
Grade 3	3 (33.3%)	0 (0%)	3 (37.5%)	
Femoropopliteal lesion, N (%)	76 (61.3%)	33 (54.1%)	43 (68.3%)	.152
TASC II classification				.978
TASC C	22 (28.9%)	9 (27.3%)	13 (30.2%)	
TASC D	46 (60.5%)	20 (60.6%)	26 (60.5%)	
CTO	22 (17.7%)	13 (21.3%)	9 (14.3%)	.430
Vessel calcification				.745
Grade 3	17 (13.7%)	8 (13.1%)	9 (14.3%)	
Grade 4	21 (16.9%)	9 (14.8%)	12 (19%)	
Infra-popliteal lesion N (%)	120 (96.8%)	60 (98.4%)	60 (95.2%)	.619
TASC II classification				.768
TASC C	36 (29%)	17 (27.9%)	19 (30.2%)	
TASC D	70 (56.4%)	34 (55.7%)	36 (57.1%)	
CTO	101 (81.5%)	51 (83.6%)	50 (79.4%)	.707
Vessel calcification				.299
Grade 3	17 (13.7%)	7 (11.5%)	10 (15.9%)	
Grade 4	27 (21.8%)	12 (19.7%)	15 (23.8%)	
Run-off vessel numbers (0/1/2/3), N				
Before EVT	31/40/37/14	16/16/21/8	15/24/16/6	.459
After EVT	1/28/63/30	0/9/35/17	1/19/28/13	.117
Median run-off vessel numbers, N				
Before EVT	1.0 (0–2)	1.0 (0–2)	1.0 (0.5–2)	.447
After EVT	2.0 (2–2.3)	2.0 (2–3)	2.0 (1–2)	.050
Occlusion of plantar arch, N (%)	46 (38.7%)	20 (33.3%)	26 (44.1%)	.311
Procedure success	113 (91.1%)	57 (93.4%)	56 (88.9%)	.565
Stenting	42 (33.9%)	15 (24.6%)	27 (42.9%)	.050
Drug coating balloon				
Femoropopliteal	19 (15.3%)	7 (11.5%)	12 (19.0%)	.357
Infra-popliteal	19 (15.3%)	11 (18.0%)	8 (12.7%)	.565
18-month clinical outcome				
Target Lesion Revascularization	18 (14.5%)	9 (14.8%)	9 (14.3%)	1.000
All-cause mortality	29 (23.4%)	8 (13.1%)	21 (33.3%)	.014

At 6 months, wound healing was achieved in 61 patients (Healing group) but not achieved in the other 63 patients (Non-healing group). The non-healing group patients were more likely to have chronic kidney disease than the Healing group (41 (65.1%) vs 28 (45.9%, respectively), *P* = .049), lower hemoglobin (9.7 g/dL (8.5-10.7) vs 10.8 (8.9- 12.9, respectively, *P* = .004), a higher Rutherford class (Rutherford category 6: 46 (73.0%) vs 24 (39.3%), respectively, *P* < .001), as well as a worse wound status (Texas Grade 3, 27 (42.9%) vs 14 (23%), respectively, *P* = .057). The arterial lesion characteristics were similar in both groups. Median runoff vessel numbers after EVT were slightly less in the Non-healing group (2.0 (1-2) vs 2.0 (2-3), *P* = .05), while the stenting rate was slightly higher in the Non-healing group (27 (42.9%) vs 15 (24.6%), *P* = .05). At the 18-month clinical follow-up, there was no significant difference in target lesion revascularization between the 2 groups, however all-cause mortality was significantly higher in the Non-healing group (21 (33.3%) vs 8 (13.1%), *P* = .014).

### 3.1 Thermography

Thermographic findings in the patients are shown in Table 3. Mean pre- and post-EVT temperature of the feet was significantly higher in the Healing group than in the Non-healing group (30.78 °C (28.94- 32.38) vs 29.42 °C (26.84- 31.38), *p* = .015; and 32.34 °C (30.48- 33.23) vs 30.96 °C (28.74- 32.48), *P* = .004, respectively). However, the temperature gain after EVT was not different between 2 groups. DIFF2 also differed significantly between the 2 groups (-0.90 °C (-1.52- -0.59) vs -1.38 °C (-1.82- -0.94), *P* = .009), but not DIFF1 (-1.12 °C (-1.70- -0.74) vs -1.14 °C (-1.68- -0.80), *P* = .605) or DIFF2 minus DIFF1 (0.10 °C (-0.26- 0.59) vs -0.14 °C (-0.68- 0.46), *P* = .07).

**Table 3:** Comparison of thermographic parameters between patients with/without wound healing or major amputation at 6 months.

	All patients N = 124	Healing in 6-mo Group N = 61	Non-Healing in 6-mo Group N = 63	*P* value
Mean pre-EVT temperature of whole foot (°C)	30.05 (28.00–31.87)	30.78 (28.94–32.38)	29.42 (26.84–31.38)	.015
Mean post-EVT temperature of whole foot (°C)	31.67 (29.30–32.80)	32.34 (30.48–33.23)	30.96 (28.74–32.48)	.004
Post- minus pre-EVT temperature of whole foot (°C)	1.30 (−0.65–3.24)	1.30 (−0.64–3.46)	1.30 (−0.78–3.12)	.994
DIFF1 (°C)	−1.13 (−1.69–0.79)	−1.12 (−1.70–0.74)	−1.14 (−1.68–0.80)	.605
DIFF2 (°C)	−1.12 (−1.78–0.69)	−0.90 (−1.52–0.59)	−1.38 (−1.82–0.94)	.009
DIFF2 minus DIFF1 (°C)	0.00 (−0.52–0.57)	0.10 (−0.26–0.59)	−0.14 (−0.68–0.46)	.070
	All patients N = 124	No-Major Amputation N = 110	Major Amputation N = 14	
Mean pre-EVT temperature of whole foot (°C)	30.05 (28.00–31.87)	30.05 (27.95–31.76)	30.03 (28.35–33.17)	.567
Mean post-EVT temperature of whole foot (°C)	31.67 (29.30–32.80)	31.72 (29.38–32.95)	31.00 (28.94–32.35)	.407
Post- minus pre-EVT temperature of whole foot (°C)	1.30 (−0.65–3.24)	1.38 (−0.63–3.66)	0.47 (−1.10–1.79)	.398
DIFF1 (°C)	−1.13 (−1.69–0.79)	−1.11 (−1.60–0.76)	−1.85 (−2.67–0.99)	.026
DIFF2 (°C)	−1.12 (−1.78–0.69)	−1.08 (−1.67–0.68)	−1.82 (−2.21–1.45)	.004
DIFF2 minus DIFF1 (°C)	0.00 (−0.52–0.57)	0.01 (−0.40–0.57)	−0.25 (−1.10–0.96)	.525

In terms of freedom from major amputation, there was no significant difference in the mean pre- and post-EVT temperature of the whole foot between the freedom from major amputation and major amputation groups. However, both DIFF1 and DIFF2 were significantly lower in the major amputation groups (-1.85 °C (-2.67- -0.99) vs -1.11 °C (-1.60- -0.76), *P* = .026 and -1.82 °C (-2.21- -1.45) vs -1.08 °C (-1.67- -0.68), *P* = .004, respectively). However, DIFF2 minus DIFF1 was not different between the 2 groups.

### 3.2 Predictors for wound healing and freedom from major amputation

Univariate Cox regression analysis revealed that DIFF2 (hazard ratio [HR] 1.46 (1.01– 2.09, *P* = .041, Table 4), mean post-EVT foot temperature (HR 1.11 (1.01– 1.21), *P* = .026), chronic kidney disease (HR 0.57 (0.34– 0.94), *P* = .027), a Rutherford classification of 6 (HR 0.43 (0.25– 0.72), *P* = .001), occluded plantar arch (HR 0.57 (0.34- 0.98), *P* = .044), along with run-off vessel numbers after EVT (HR 1.72 (1.19- 2.51), *P* = .004) were all predictors for wound healing occuring at 6 months. However, only a Rutherford classification of 6 (HR 0.46 (0.26– 0.82), *P* = .008), occluded plantar arch (HR 0.58 (0.33– 1.01), *P* = .055) and run-off vessel numbers after EVT (HR 1.65 (1.09- 2.51), *P* = .018) remained as significant predictors for 6-month wound healing in multivariate Cox regression analysis.

**Table 4:** Predictors for wound healing.

	Univariate analysis			Multivariate analysis		
	HR	95% CI	*P* value	HR	95% CI	*P* value
Age (yr)	1.00	(0.98–1.02)	.678			
Gender (Male vs Female)	1.49	(0.87–2.57)	.147			
Current smoking	1.33	(0.57–3.09)	.510			
Current dialysis	0.70	(0.40–1.22)	.211			
Chronic kidney disease	0.57	(0.34–0.94)	.027	0.62	(0.36–1.07)	.086
Hemoglobin	1.12	(1.02–1.24)	.020	1.02	(0.90–1.15)	.788
Rutherford classification (6 vs 5)	0.43	(0.25–0.72)	.001	0.46	(0.26–0.82)	.008
University of Texas classification						
Grade 0 as reference						
Grade 1	0.57	(0.26–1.26)	.166			
Grade 2	0.50	(0.21–1.20)	.121			
Grade 3	0.35	(0.15–0.84)	.019			
Infection	0.78	(0.47–1.28)	.326			
Occluded plantar arch	0.57	(0.34–0.98)	.044	0.58	(0.33–1.01)	.055
Run-off vessel number after EVT	1.72	(1.19–2.51)	.004	1.65	(1.09–2.51)	.018
Prostaglandin E1 use	1.21	(0.70–2.08)	.492			
Femoropopliteal drug-coating balloon	0.57	(0.26–1.25)	.158			
Infra-popliteal drug-coating balloon	1.02	(0.53–1.95)	.961			
Mean pre-EVT temperature of whole foot	1.07	(0.98–1.17)	.111			
Mean post-EVT temperature of whole foot	1.11	(1.01–1.21)	.026	1.07	(0.96–1.19)	.218
DIFF1	1.10	(0.92–1.31)	.310			
DIFF2	1.46	(1.01–2.09)	.041	1.06	(0.73–1.52)	.774

In terms of major amputation, being currently on dialysis (HR 4.07 (1.36– 12.21), *P* = .012, Table 5), hemoglobin (HR 0.65 (0.46– 0.91), *P* = .014), Rutherford classification of 6 (HR 4.87 (1.09–21.78), *P* = .038), infection (HR 12.07 (1.58– 92.27), *P* = .016) and DIFF2 (HR 0.50 (0.31– 0.83), *P* = .007) were predictors for major amputation upon univariate analysis. However, only hemoglobin (HR 0.68 (0.47– 0.99), *P* = .047), infection (HR 11.78 (1.40– 98.95), *P* = .023) and DIFF2 (HR 0.51 (0.27–0.98), *P* = .045) were independent predictors for 6-month major amputation in multivariate analysis.

**Table 5:** Predictors for major amputation.

	Univariate analysis			Multivariate analysis		
	HR	95% CI	*P* value	HR	95% CI	*P* value
Age (yr)	0.99	(0.96–1.03)	.721			
Gender (Male vs Female)	0.84	(0.29–2.43)	.751			
Current smoking	1.02	(0.13–7.79)	.986			
Current dialysis	4.07	(1.36–12.21)	.012	2.30	(0.75–7.10)	.146
Chronic kidney disease	3.30	(0.92–11.85)	.067			
Hemoglobin	0.65	(0.46–0.91)	.014	0.68	(0.47–0.99)	.047
Rutherford classification (6 vs 5)	4.87	(1.09–21.78)	.038	1.36	(0.29–6.45)	.698
University of Texas classification						
Grade 1 as reference						
Grade 2	3.00	(0.27–33.09)	.370			
Grade 3	13.05	(1.68–101.20)	.014			
Infection	12.07	(1.58–92.27)	.016	11.78	(1.40–98.95)	.023
Occluded plantar arch	1.60	(0.56–4.57)	.378			
Run-off vessel number after EVT	0.87	(0.42–1.78)	.693			
Prostaglandin E1 use	0.20	(0.03–1.53)	.121			
Femoropopliteal drug-coating balloon	0.43	(0.06–3.26)	.412			
Infra-popliteal drug-coating balloon	0.38	(0.05–2.92)	.353			
Mean pre-EVT temperature of whole foot	1.08	(0.90–1.29)	.409			
Mean post-EVT temperature of whole foot	0.98	(0.82–1.16)	.795			
DIFF1	0.91	(0.79–1.06)	.229			
DIFF2	0.50	(0.31–0.83)	.007	0.51	(0.27–0.98)	.045

**Figure 2:**
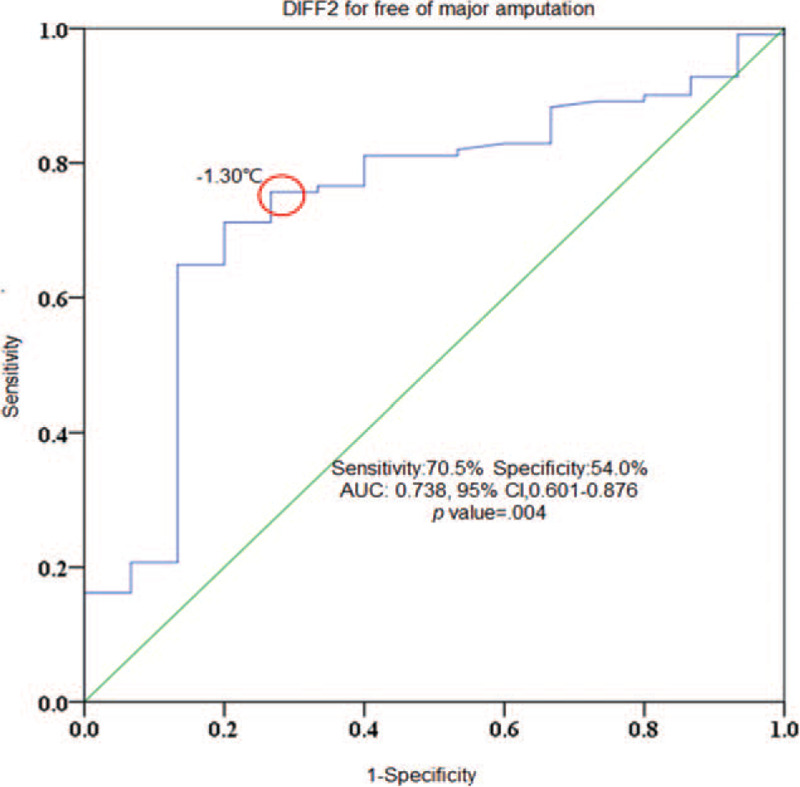
Receiver operating characteristic curve analysis for DIFF2 in predicting freedom from major amputation. At a DIFF2 value of ≧ -1.3 °C, it predicted freedom from major amputation with a sensitivity of 70.5%, specificity of 54.0%, positive predictive value of 59.7%, and negative predictive value of 65.4% (area under curve 0.738, 95% confidence interval [CI],0.601-0.876; *P* = .004).

The best cutoff value of DIFF2 for predicting freedom from major amputation was determined by ROC curve analysis and is shown in Figure 2. A DIFF2 ≧ -1.3 °C best predicted freedom from major amputation with an area under the curve of 0.738 (0.601–0.876), sensitivity of 70.5%, specificity of 54.0%, positive predicting value of 59.7%, and a negative predicting value of 65.4%, *P* value = .004).

### 3.3 Predictors for mortality

Age (HR 1.03 (1.00-1.07), *P* = .037), BMI (HR 0.89 (0.81-0.97), *P* = .008, Table 6), currently on dialysis (HR 2.69 (1.29-5.61), *P* = .008), stenting(HR 2.28 (1.09-4.76), *P* = .028), mean pre-EVT temperature of whole foot (HR 0.89 (0.80-0.99), *P* = .039) and mean post-EVT temperature of whole foot (HR 0.88 (0.78-0.99), *P* = .029) were predictors for mortality in univariate analysis. Only age (HR 1.05 (1.01-1.09), *P* = .013) and currently on dialysis (HR 4.38 (1.88-10.18), *P* = .001, were independent predictors for mortality in multivariate analysis. However, either DIFF1 (HR 1.11 (0.84-1.47), *P* = .474) or DIFF2 (HR 0.84 (0.58-1.23), *P* = .365) was not predictor for mortality upon in univariate or multivariate analysis.

**Table 6:** Predictors for mortality.

	Univariate analysis				Multivariate analysis			
	HR	95% CI	*P* value	HR	95% CI	*P* value
Age (yr)	1.03	(1.00–1.07)	.037	1.05	(1.01–1.09)	.013
Gender (male vs female)	0.85	(0.40–1.84)	.687			
Body mass index, kg/m^2^	0.89	(0.81–0.97)	.008	0.92	(0.82–1.03)	.154
Chronic lung disease	1.80	(0.73–4.43)	.203			
Congestive heart failure	1.88	(0.91–3.90)	.090			
Coronary artery disease	1.69	(0.81–3.54)	.161			
Currently on dialysis	2.69	(1.29–5.61)	.008	4.38	10.18)	.001
GFR (mL/min)	0.99	(0.98–1.00)	.098			
Low density lipoprotein, mg/dL	0.99	(0.98–1.00)	.103			
Rutherford classification (6 vs 5)	2.08	(0.91–4.79)	.083			
Stenting	2.28	(1.09–4.76)	.028	1.56	(0.64–3.81)	.328
Femoropopliteal drug-coating balloon	1.07	(0.37–3.07)	.906			
Infra-popliteal drug-coating balloon	0.34	(0.08–1.42)	.138			
Mean pre-EVT temperature of whole foot	0.89	(0.80–0.99)	.039	0.96	(0.87–1.06)	.391
Mean post-EVT temperature of whole foot	0.88	(0.78–0.99)	.029			
DIFF1	1.11	(0.84–1.47)	.474			
DIFF2	0.84	(0.58–1.23)	.365			

### 3.4 Time to wound healing and freedom from major amputation

**Figure 3:**
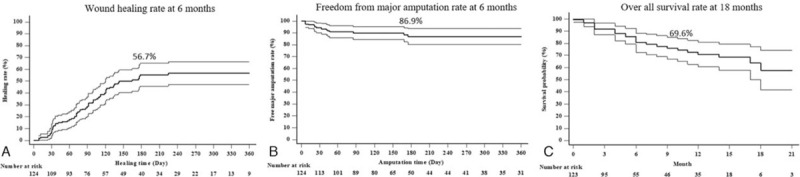
Kaplan–Meier survival curves of post-EVT wound healing, freedom from major amputation and overall survival in all CLI patients. Panel A: Overall wound healing was 28.4% and 56.7% at 3 and 6 months respectively; Panel B: Freedom from major amputation was 89.9% and 86.9% at 3 and 6 months respectively; Panel C: Overall survival was 69.6% and 62.6% at 12 and 18 months respectively.

The Kaplan-Meier survival curves for wound healing, freedom from major amputation and patient survival are shown in Figure 3. The wound healing rate was 28.4% and 56.7% at 3 months and 6 months respectively, whereas the time to median wound healing was 164 days. Eighteen (18) out of 124 (14.5%) patients underwent re-intervention to achieve freedom from ischemic symptoms during the 18-month follow-up period. The freedom from major amputation rate was 89.9% and 86.9% at 3 months and 6 months, respectively, whereas the over-all survival was 69.6% and 62.6% at 12 months and 18 months, respectively.

## 4 Discussion

The major findings taken from the present study are

(1) foot temperatures were significantly higher in the patients showing wound healing during follow up;
(2) the derived thermographic measurements (DIFF2 and/or DIFF1) were significantly lower in the Non-healing patients and patients suffering from major amputation;
(3) the mean post-EVT foot temperature and DIFF2 were univariate analysis predictors for wound healing but not independent predictors in multivariate analysis; and
(4) the derived thermographic parameter DIFF2 was an independent predictor for freedom from major amputation in both uni- and multi-variate analyses.

To the best of our knowledge, this is the first study to indicate that infrared thermography could be used to predict freedom from major amputation during a 6-month follow-up in patients with CLI who had received contemporary state-of-the-art EVT.

Despite successful revascularization therapy for CLI, wound healing and freedom from major amputation could not be achieved in some of the patients. Post-interventional wound healing is a complex process and affected by multiple factors, including incomplete revascularization, persistent deep or extensive infections, poor wound care or vessel reocclusion after initial opening.^[4,19]^ Reflected in our study results is that wound healing was predicted by the Rutherford classification of 6, as well as fewer run-off vessels to the foot, and tended to be related to plantar arch occlusion in multi-variate analysis; whereas chronic renal disease, a lower hemoglobin count, mean post-EVT foot temperature and DIFF2 were also significant predictors in uni-variate analysis. In terms of need for major amputation, a lower hemoglobin count, foot infection and DIFF2 were independent predictors in multi-variate analysis; and despite currently undergoing dialysis, a Rutherford classification of 6, along with a high grade Texas classification were also predictors in uni-variate analysis.

The current tools for predicting wound outcomes all have significant limitations. Skin perfusion pressure (SensiLase^TM^ PAD 3000 skin perfusion pressure system, [SPP]) and transcutaneous pressure of oxygen (TCPO2) (PeriFlux 6000, TCPO2) are 2 currently available tools for the evaluation of microcirculation.^[1,6,7,20–22]^ Castronuovo et al reported that the measurement of SPP was useful for the evaluation of the microcirculatory function after EVT in patients with CLI, and that SPP values ≥40 mm Hg were associated with wound healing in critically ischemic limbs. SPP is useful and can be carried out with simplicity and reproducibility after EVT. However, both SPP and TCPO2 examinations are costly, particularly when repetitive tests are needed for a single CLI patient. Laser doppler technology, i.e. laser doppler fluxmetery, also has been used to assess limb ischemia.^[23]^ Similar to percutaneous oxymetry, this technique measures the blood flow responses to stress at the small skin area. There was report showing it was more sensitive and accurate in predicting poor response to revascularization and subsequent amputation in patients with severe leg ischemia.^[23,24]^ However, skin contact is needed.

Although the most widely used test for assessment of patients at risk for PAD is the ABI, the test has substantial limitations in individuals with diabetes and arterial calcification.^[9,20]^ Alternative arterial studies, such as the Toe-Brachial Index and pulse volume recording measurement, have been suggested for detecting PAD in individuals who are at risk for lower extremity PAD and have an ABI greater than 1.30.^[9]^ Additionally, echo-Doppler is a less costly and safe technique which may be implemented. In expert hands, it can reliably show the main anatomic characteristics necessary in order to undertake revascularization. However, it is excessively dependent on the operator and is poorly reliable and time consuming with regards to infrapopliteal vessels.^[8]^ However little information surrounding microcirculation relating to wound healing could be obtained by ABI, toe brachial index, PVR and echo-Doppler after EVT. In contrast, infrared thermography is very cost-effective and has the advantages of being noninvasive, fast, reliable, non-contacting, and capable of producing multiple recordings at short time intervals. One of the major advantages of thermography is that the technology allows all limbs to be assessed in 1 test. A further advantage is that this test does not involve any direct contact with the patient's skin and is completely acceptable to the patient. The lack of device contact with the patient has significant advantages with regards to infection control. The procedure can also be repeated several times without causing patients any distress or exposing them to any possible risk. A previous study showed that thermography could detect improvement in perfusion as reflected by an increase in skin temperature after endovascular intervention.^[13]^ Assessing temperature over the metatarsal area of the foot, and in the heels as well as in the shins, was shown to be effective in detecting changes after intervention; whereas knowing the temperature in the toes was shown to be less useful as they were exposed parts of the body and affected by ambient temperatures.^[25]^ One study showed the thermal gradient (>0.4 °C) between toes as measured by the interdigital anisothermal test in a diabetic foot in contrast to the control subject.^[26]^ In the current study, we used both a physiological and rational approach by adopting angiosome-based thermographic measurements and measuring 5 zones instead of only 1 target zone as infection across more than 1 zone was seen in more than half of our study population. We also used derived thermographic parameters, DIFF1 and DIFF2, to better find a tool to help the clinicians in predicting wound outcome following EVT. The less of a thermal gradient indicated a better outcome for limb salvage. In our study, we demonstrated that DIFF2 worked as an independent predictor for freedom from major amputation after EVT for CLI. In terms of non-invasive measurement, Laser Doppler scan (PeriScan, PIM II, Perimed AB, Järfälla, Sweden) uses a moving laser (670 nm) beam to non-invasively detect superficial tissue blood flow and has also been used to assess hand ischemia.^[27]^ Using different scan modes, it could accurately measure blood flow in an area of interest or the blood flow over time at a single point using the Doppler principle. The flow map images of laser doppler is generated by the matrix scan of a single laser beam over the area and made in several minutes, in contrast to the infrared thermography images made over more-extended area of interest and generated instantly by the camera. Therefore, the thermography method is easier to use, more time-saving, and better in spatial and temporary resolution. Given these merits, we did believe thermography images might be a better way to assess the temperature effect of the diminished blood flow at the baseline and the impacts of the PTA, despite both methods could be used in clinical assessment after PTA.

Furthermore, the clinical impact of peripheral limb ischemia has been underestimated for long time. It is associated not only with risk of critical ischemia and amputations, but also with vascular complications in remote vascular beds. This is due both to the presence of common underlying risk factor or pathophysiological mechanisms and to a paracrine effect through the release of soluble factors into the blood stream that may exert their effect in different vascular districts, such as mediators of inflammation, reactive oxygen species.^[28]^ Indeed, it is known that limb ischemia can negatively influence vascular remodeling in other districts, such as coronary and cerebral arteries.^[29–32]^ Very recently, it was shown that the microRNA-16 is indeed able to modulate endothelial function and vascular remodeling in carotid arteries upon induction of ischemia of peripheral limbs.^[33]^ Whether successful revascularization by PTA could help revert these negative remove effects await future studies.

Our Study has several limitations. First, this study was performed in a single research center with a relatively small study population. Further multicenter studies with large numbers of patients are required in order to confirm the present study results. Second, wound healing and freedom from major amputation are complex and affected by multiple factors such as treatments offered by plastic surgeons, simultaneous superficial or deep infections, and vessel re-occlusion after initial opening. The effect of opening occluded vessels may be mitigated by either extensive infections refractory to antibiotics or debridement. Therefore, simply measuring the foot thermography may not translate into the final clinical end result of wound healing. Third, the use of Promostan (Prostaglandin E1) could have interfered with our result. However, only a small percentage (28.2%) of the study participants were given this vasodilator, and there were no statistically significant differences between the Healing and Non-healing subgroups, and major amputation and no major amputation subgroup patients who were given this medication.

In Conclusion, wound healing and freedom from major amputation in CLI patients is complex, challenging and demands special attention. Infrared thermography has been shown to be effective in identifying changes in the skin temperature in the CLI patients undergoing EVT. The direct and derived thermographic measurements were found to be associated with wound healing, but not an independent predictor for it. The derived thermographic measurement of DIFF2 could be used as an independent predictor for 6-month freedom from major amputation in clinical practice. Further, larger studies are still required in order to confirm the current findings taken from our study.

## Acknowledgments

We thank the staff of the Biostatistics Task Force of Taichung Veterans General Hospital, Taichung, Taiwan, ROC for their excellent assistance in data analysis.

## Author contributions

**Conceptualization:** Wei-Chun Chang, Chi-Yen Wang, Yutsung Cheng, Yu-Po Hung, Tzu-Hsiang Lin, Wei-Jhong Chen, Chieh-Shou Su, Chiann-yi Hsu, Tsun-Jui Liu, Wen Lieng Lee.

**Data curation:** Wei-Chun Chang, Chi-Yen Wang, Yutsung Cheng, Yu-Po Hung.

**Formal analysis:** Wei-Chun Chang, Chi-Yen Wang, Chiann-yi Hsu.

**Funding acquisition:** Wei-Chun Chang.

**Investigation:** Wei-Chun Chang.

**Methodology:** Wei-Chun Chang, Chieh-Shou Su, Chiann-yi Hsu, Tsun-Jui Liu, Wen Lieng Lee.

**Project administration:** Wei-Chun Chang.

**Resources:** Wei-Chun Chang, Yutsung Cheng, Yu-Po Hung, Tzu-Hsiang Lin, Wei-Jhong Chen, Chieh-Shou Su.

**Software:** Wei-Chun Chang, Chiann-yi Hsu.

**Supervision:** Tsun-Jui Liu, Wen Lieng Lee.

**Validation:** Tsun-Jui Liu.

**Visualization:** Wen Lieng Lee.

**Writing – original draft:** Wei-Chun Chang.

**Writing – review & editing:** Tsun-Jui Liu, Wen Lieng Lee.
